# Chemical and Transcriptomic Analyses of Leaf Cuticular Wax Metabolism in *Ammopiptanthus mongolicus* under Osmotic Stress

**DOI:** 10.3390/biom14020227

**Published:** 2024-02-16

**Authors:** Batu Sumbur, Minqi Zhou, Tashi Dorjee, Jie Bing, Sijia Ha, Xiaojing Xu, Yijun Zhou, Fei Gao

**Affiliations:** 1 Key Laboratory of Mass Spectrometry Imaging and Metabolomics, Minzu University of China, National Ethnic Affairs Commission, Beijing 100081, China; songbuerbatu@muc.edu.cn (B.S.); 21400283@muc.edu.cn (M.Z.); 20400268@muc.edu.cn (T.D.); 22302467@muc.edu.cn (S.H.); xuxiaojing@muc.edu.cn (X.X.); 2 Key Laboratory of Ecology and Environment in Minority Areas, Minzu University of China, National Ethnic Affairs Commission, Beijing 100081, China; 3 College of Life and Environmental Sciences, Minzu University of China, Beijing 100081, China; 4 College of Life Sciences, Beijing Normal University, Beijing 100080, China; bingjie@bnu.edu.cn

**Keywords:** *Ammopiptanthus mongolicus*, cuticular wax, alkanes, osmotic stress, transcriptome

## Abstract

Plant cuticular wax forms a hydrophobic structure in the cuticle layer covering epidermis as the first barrier between plants and environments. *Ammopiptanthus mongolicus*, a leguminous desert shrub, exhibits high tolerances to multiple abiotic stress. The physiological, chemical, and transcriptomic analyses of epidermal permeability, cuticular wax metabolism and related gene expression profiles under osmotic stress in *A. mongolicus* leaves were performed. Physiological analyses revealed decreased leaf epidermal permeability under osmotic stress. Chemical analyses revealed saturated straight-chain alkanes as major components of leaf cuticular wax, and under osmotic stress, the contents of total wax and multiple alkane components significantly increased. Transcriptome analyses revealed the up-regulation of genes involved in biosynthesis of very-long-chain fatty acids and alkanes and wax transportation under osmotic stress. Weighted gene co-expression network analysis identified 17 modules and 6 hub genes related to wax accumulation, including 5 enzyme genes coding KCS, KCR, WAX2, FAR, and LACS, and an ABCG transporter gene. Our findings indicated that the leaf epidermal permeability of *A. mongolicus* decreased under osmotic stress to inhibit water loss via regulating the expression of wax-related enzyme and transporter genes, further promoting cuticular wax accumulation. This study provided new evidence for understanding the roles of cuticle lipids in abiotic stress tolerance of desert plants.

## 1. Introduction

Terrestrial plants are inevitably confronted with multiple abiotic stresses such as drought, high salinity, extreme temperatures, and intense radiation throughout their life cycle. These threats compelled plants to evolve a repertoire of physiological and metabolic mechanisms to sustain their vital functions in adverse environmental conditions [1,2]. Abiotic stresses, like water deficit and high salinity, lead to excessive water loss from plants to the external environment, which inhibits plant development and poses a persistent challenge hampering agricultural productivity [3,4]. Therefore, it is becoming more urgent to elucidate the mechanisms of plant tolerance to osmotic stress and construct crop varieties with enhanced osmotic resistance. In recent years, a series of knowledge regarding the molecular mechanisms of stress tolerance in plants has been acquired through investigations on model plants such as *Arabidopsis* and *Oryza sativa* [5]. However, the exploration of stress adaptation mechanisms in plant species which have endured in severe environment for a long time remains insufficient.

The plant cuticle lipids mainly encompass the aliphatics constituting the outermost layer of plant tissues, which directly interact with the external environment. In addition to the wax and cutin layers enveloping the surface of nutritive organs including leaves and stems, plant cuticle lipids also extend to the suberin which is deposited between the primary cell wall and the plasma membrane of inner layer in roots, as well as the lipids existing on the surface of calluses, pollens, and seed coats [6,7]. Cuticle lipids have been observed that formed a multi-layer structure on the leaf epidermis: the epicuticular wax film composed of crystalline mixtures is the outermost layer, and beneath that is the cuticular layer composed of various polymerized cutin monomers, with intracuticular wax embedded, which is connected with the inside epidermal cell wall [8]. To date, the composition of cuticular wax in various plants has been identified by mass-spectrometry. Plant cuticular wax is primarily composed of very-long-chain saturated fatty acids (VLCFAs) and derivatives such as alkanes, aldehydes, alcohols, alkyl esters, ketones, and diketones. The carbon chain lengths of these aliphatics generally range from C_20_ to C_37_ [9]. Previous studies have revealed that the composition and content of cuticular wax varied in different plant organs. For instance, research on tomato epidermal wax revealed that the total cuticular wax on the stem of tomato had a five-fold higher content compared to that of the leaf [10]. Tissue wax analysis of *Thellungiella salsuginea* suggested that alkanes, ketones, and secondary alcohols (2-alcohols) were the main components in the cuticular wax of leaves and stems, while alkanes and acids dominated in flowers [11]. The biosynthesis of plant cuticular wax involves a complex biochemical reaction network, with multiple branching pathways included. The precursors for wax synthesis are C_16_ and C_18_ saturated fatty acids, which are synthesized in the cytoplasmic matrix. Subsequently, these precursors are transported to the endoplasmic reticulum (ER) for further elongation of carbon chains, leading to the production of very-long-chain saturated fatty acids (VLCFAs). Following this, VLCFAs are further transformed into multiple wax components through the acyl reduction pathway (for 1-alcohol and ester production) and decarboxylation pathway (for aldehyde, alkane, 2-alcohol and ketone production) involving multi-step bio-reactions, respectively [12]. After the production in ER, wax components are transported across the cytoplasmic membrane and the cell wall via specific transport proteins and are ultimately secreted on the plant tissue surface, forming the wax layer [13].

Cuticular wax evolved from long-term plant adaptation to the environment. The wax layer, in conjunction with the cuticle layer, constitutes the first barrier for plants against external threats. Cuticular wax was reported to play an important role in plant response to abiotic stresses [12]. Abiotic stresses, such as drought and high salinity, lead to excessive water loss in plants. Cuticular wax mixture, functioning as a hydrophobic layer on the surface of plant tissues, was identified as a crucial structure in maintaining water retention and responding to osmotic stress [14]. Previous studies have indicated the significant accumulation of cuticular wax under drought stress in multiple plant species, such as *Arabidopsis*, tobacco, and wheat [13,15,16]. Furthermore, some functional genes which directly affected the synthesis of wax components have been isolated, thereby participating in the drought response mechanism. For example, *AtCER1* (*ECERIFERUM1*) isolated from *Arabidopsis* were revealed; they encoded an aldehyde decarboxylase protein. Overexpression of *AtCER1* promoted the biosynthesis of alkanes in cuticular wax and enhanced the drought tolerance of *Arabidopsis* seedlings [17]. A total of three fatty acyl-coenzyme A reductase (FAR) coding genes, i.e., *TaFAR2*, *TaFAR3*, and *TaFAR4*, were identified from wheat, which directly catalyzed the production of C_18_, C_24_ and C_28_ saturated primary alcohols (1-alcohols) in wax, further participating in wheat response to drought stress [18]. An *Arabidopsis cer3* mutant exhibited a significant decrease in leaf cuticular wax content and a reduced tolerance to drought, indicating that *AtCER3* participated in drought response by affecting wax accumulation [19]. The cuticular wax layer was also reported as a protector for plants against low-temperature stress. Previous studies have unveiled the accumulation of wax in plants exposed to cold environments. For example, research on tea plants in different seasons demonstrated that the total wax content and number of wax components enhanced, and wax composition significantly varied during leaf growth from June to September [20]. In addition, previous studies have demonstrated that some plant species such as cucumber and common bean had a significant increase in total wax content in response to UV radiation [14].

*Ammopiptanthus mongolicus* is an evergreen, broad-leaved shrub belonging to the genus *Ammopiptanthus*, Leguminosae family. The genus *Ammopiptanthus* has origins traced back to the Tertiary Period and had withstood the drastic environmental change caused by the Himalayan uplift [21]. *A. mongolicus* inhabit arid desert regions and are exposed to harsh environmental challenges for prolonged times, including drought, extreme temperature, and intense radiation, which caused this shrub to develop a remarkable tolerance to multiple abiotic stresses. *Ammopiptanthus* species are used by local inhabitants as medicines to treat frostbite, chronic rheumatoid arthritis, and disperse petechia. Some bioactive components, such as quinolizidine alkaloids, flavonoids, and resveratrol, have also been identified [22]. In recent years, researchers have taken interest in *A. mongolicus* as a valuable resource for investigating the molecular mechanisms of stress tolerance in woody plants. Several omics analyses have been conducted to unveil the abiotic stress response mechanisms of *A. mongolicus*, and some stress response-related genes have been isolated [23,24,25]. It is speculated that cuticular wax plays important roles in drought stress response in *A. mongolicus*, which has inhabited the desert for a long time. However, the analysis of alterations in cuticular wax and related gene expression under water deficit stresses has not been reported so far.

In the present study, the analyses of cuticular wax metabolites and transcriptome of *A. mongolicus* leaves under osmotic stress were executed, and the major responded wax components and related biosynthesis and transportation pathways were identified. Significantly responding enzyme and transporter genes were revealed from transcriptome data, and key genes affecting wax accumulation were also identified. This study provides new evidence for understanding the roles of cuticle lipids in stress tolerance mechanisms of woody plants growing in desert habitats.

## 2. Materials and Methods

### 2.1. Plant Materials and Stress Treatment

The seeds of *A. mongolicus* were collected from Otog county, Inner Mongolia Autonomous Region, China. The seeds were surface sterilized using 70% (*v*/*v*) ethanol for 1 min, followed by bleaching (10%) for 6 min and washing in sterile water for 5 min, and then planted in a 30 cm diameter pot containing a 3:1 (*v*/*v*) mixture of vermiculite and perlite. Seedlings were grown in a growth chamber under 120 µmol m^−2^ s^−1^ photosynthetic photon flux density, with a photoperiod cycle of 16 h of light and 8 h of dark, at approximately 25 °C and 35% relative humidity. The seedlings were watered every 4 d with a half-strength Hoagland solution [26]. Eight weeks after germination, seedlings with similar growth were randomly divided into six groups, each containing approximately 50 seedlings. Five groups of them were irrigated with a 20% PEG6000 solution for 6 h, 24 h, 72 h, 7 d, and 14 d for osmotic stress treatment. The other group (control) continued to grow under the initial conditions. The sampled tissues were snap-frozen in liquid nitrogen and placed at −80 °C for metabolite analysis and RNA extraction.

### 2.2. Measurement of the Water Loss Rate of Leaves

To measure the water loss rate of excised leaves, the seedlings were placed in the dark for at least 4 h so as to close the stomata and stop the stomatal transpiration prior to measurement. The sampled leaves were immediately placed in water (in the dark) for 30 min soak to equilibrate leaf water contents. Then, the leaves were shaken gently and rapidly blotted dry to remove all visible excess water and suspended from a string in the dark with gentle air circulation. Leaf weights were recorded every 30 min over a 300 min period, and water loss rates were expressed as a percentage of the initial water-saturated fresh weight (FW). Three independent biological replicates were used for all assays.

### 2.3. Measurement of the Chlorophyll Efflux Rate of Leaves

The measurement of the chlorophyll efflux rate of leaves was conducted according to a previous method [27]. Briefly, the seedlings were watered and placed in the dark for at least 4 h prior to measurement. Excised leaves were immersed in 80% ethanol in glass vials covered by aluminum foil and agitated gently on a shaker in the dark. Aliquots of a 1 mL extracting solution were removed every 20 min over a 200 min period, till 48 h after initial immersion, at which time all chlorophyll was essentially extracted (leaves became fully gray). The content of chlorophyll was quantified by measurement of absorption at 647 and 664 nm as absorption maxima of chlorophyll b and chlorophyll a, respectively, as previously described [27]. The chlorophyll efflux rates of different time points were calculated as percentages of the total chlorophyll content. Three independent biological replicates were used for all assays.

### 2.4. Measurement of the Photosynthesis-Related Gas Exchange Indexes

The gas exchange indexes related to photosynthesis of leaves, including the net photosynthetic rate (P_n_), the transpiration rate (T_r_), stomatal conductance (G_s_), and intercellular CO_2_ concentration (C_i_), were measured using a LI-6400XT Portable Photosynthesis System (LI-COR, Lincoln, NE, USA) at a constant 400 μmol mol^−1^ CO_2_ and a luminous intensity of 1200 μmol m^−2^ s^−1^ as the saturated photosynthetic photon flux density [28]. Three independent biological replicates were used for all assays. 

### 2.5. Analyses of Leaf Cuticular Wax

The extraction and analysis of leaf cuticular wax were conducted according to previous methods [29]. The wax components were analyzed using an Agilent 6890N-5975i Electron impact gas chromatography/mass spectrometry system (GC-MS) (Agilent, Santa Clara, CA, USA) as previously described [29]. The wax content was calculated as the amount per unit of leaf surface area (μg/cm^2^). Three independent biological replicates were used for all assays.

### 2.6. Transcriptome Analyses

The TRIzol reagent was used to extract total RNA of *A. mongolicus* leaves according to the manufacturer’s protocol (Invitrogen, Burlington, ON, Canada), and DNA contamination was removed by DNase. RNA integrity was assessed using an Agilent 2100 Bioanalyzer (Agilent, Santa Clara, CA, USA), and samples with RNA Integrity Number (RIN) ≥ 7 were used to construct the cDNA library. The eligible libraries were sequenced on an Illumina HiSeq4000 platform (Illumina, Foster, CA, USA). The clean reads obtained were mapped to sequences of a previously reported *Ammopiptanthus* genome [30] using HISAT 2.1.0 with default parameters. All clean data were submitted to NCBI SRA with accession number range from SRR26384053 to SRR26384070 (included in Bioproject: PRJNA1027962). The expression level of each transcript was quantified as fragments per kilo-base of transcript per million mapped fragments (FPKM). Differentially expressed genes (DEGs) were identified using DEseq2 v1.38.3, and the criteria for defining DEGs include the fold change of ≥2 or ≤0.5 and *p*. adjustment of <0.05 (*p*-value after Benjamini–Hochberg adjustment). Three independent biological replicates were performed for each group. An R package was adopted to conduct the weighted gene co-expression network analysis (WGCNA) with default parameters. Correlation networks between hub genes were plotted using Cytoscape 3.10.0 with default parameters [31].

### 2.7. qRT-PCR Analysis

Reverse transcription and qRT-PCR were performed according to the previously described method [32]. The eIF1 gene was used as the reference for qRT-PCR analysis of *A. mongolicus* genes [33]. All the primer sequences are listed in Table S1. Three independent biological replicates were performed for each group and three technical replicates were performed for each sample. Gene expression levels were calculated using the 2^−ΔΔCt^ method [34].

### 2.8. Statistical Methods

For physiological measurements, wax analyses, and qRT-PCR analyses in the present study, the least significant difference (LSD) and DunCan Multiple Range test (DMRT), performed using SPSS 24.0 software (IBM, Armonk, NY, USA), were used to conduct multiple comparisons with default parameters. Three independent biological replicates were used for all assays.

## 3. Results

### 3.1. The Epidermal Permeability and Photosynthetic Characteristics of A. mongolicus Leaves under Osmotic Stress

**Figure 1:**
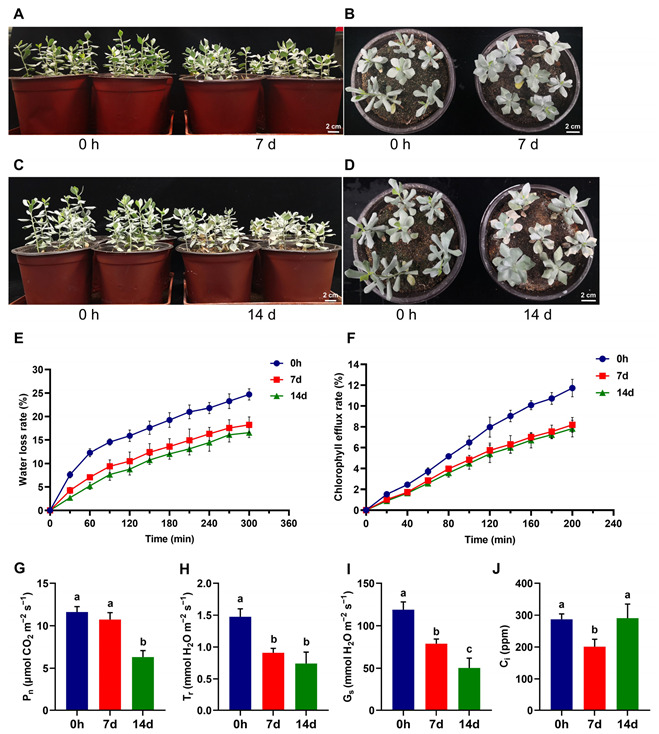
The effect of osmotic stress on morphological, physiological, and photosynthetic characteristics of *A. mongolicus* seedlings. (**A**,**B**) The morphological comparison between 7-day-osmotic-stressed and non-stressed seedlings. (**C**,**D**) The morphological comparison between 14-day-osmotic-stressed and non-stressed seedlings. Bars = 2 cm. (**E**) The water loss rates of excised leaves during a 300 min period. The water loss rate at each time point was expressed as a percentage of the initial water-saturated fresh weight. (**F**) The chlorophyll efflux rates of excised leaves during a 200 min period. The chlorophyll efflux rate at each time point was expressed as a percentage of the total chlorophyll extracted using 80% ethanol. (**G**–**J**) The photosynthetic characteristics of *A. mongolicus* leaves. (**G**) Net photosynthetic rate (P_n_). (**H**) Transpiration rate (T_r_). (**I**) Stomatal conductance (G_s_). (**J**) Intercellular CO_2_ concentration (C_i_). Each experiment was performed in three independent biological replicates. LSD and DMRT were used to conduct multiple comparisons. Lowercase letters (a–c) above columns represent the different homogeneous subsets according to multiple comparisons.

Each 45 d *A. mongolicus* seedling typically possessed 8 to 10 leaves, which exhibited white and green colors. Following a 7-day period of osmotic stress, there was no significant difference observed for the growth of seedlings compared to those without stress treatment (Figure 1A,B). As osmotic stress persisted for 14 d, a few seedlings exhibited the localized whitening in part of leaves, indicating adverse effects due to osmotic stress (Figure 1C,D).

We further analyzed the alteration of leaf epidermal permeability of *A. mongolicus* seedlings under osmotic stress. The measurement of water loss rate revealed that during a continuous 300 min measurement, the water loss rates of excised leaves subjected to osmotic stress for 7 and 14 d were consistently lower than those of non-stressed leaves at each time point (Figure 1E). The chlorophyll efflux assays demonstrated that both groups of leaves under osmotic stress exhibited lower chlorophyll efflux rates at all time points compared to non-stressed leaves during a 200 min assessment (Figure 1F). These results indicated a decrease in the epidermal permeability of *A. mongolicus* leaves under osmotic stress.

The gas exchange characteristics related to the photosynthesis of *A. mongolicus* leaves under osmotic stress were also analyzed, including the net photosynthetic rate (P_n_), the transpiration rate (T_r_), stomatal conductance (G_s_), and intercellular CO_2_ concentration (C_i_). Under saturated light intensity, the P_n_ value of leaves subjected to 7-day osmotic stress exhibited no significant alteration compared to that of non-stressed leaves (Figure 1G). Meanwhile, the values of T_r_, G_s_, and C_i_ were significantly reduced compared to the values of the control group (Figure 1H–J). For seedling leaves under 14-day osmotic stress, P_n_, T_r_, and G_s_ all demonstrated significant decreases compared to non-stressed leaves (Figure 1G–I), while the C_i_ value showed non-significant change (Figure 1J).

### 3.2. The Composition of Cuticular Wax of A. mongolicus Leaves under Control Conditions

**Figure 2:**
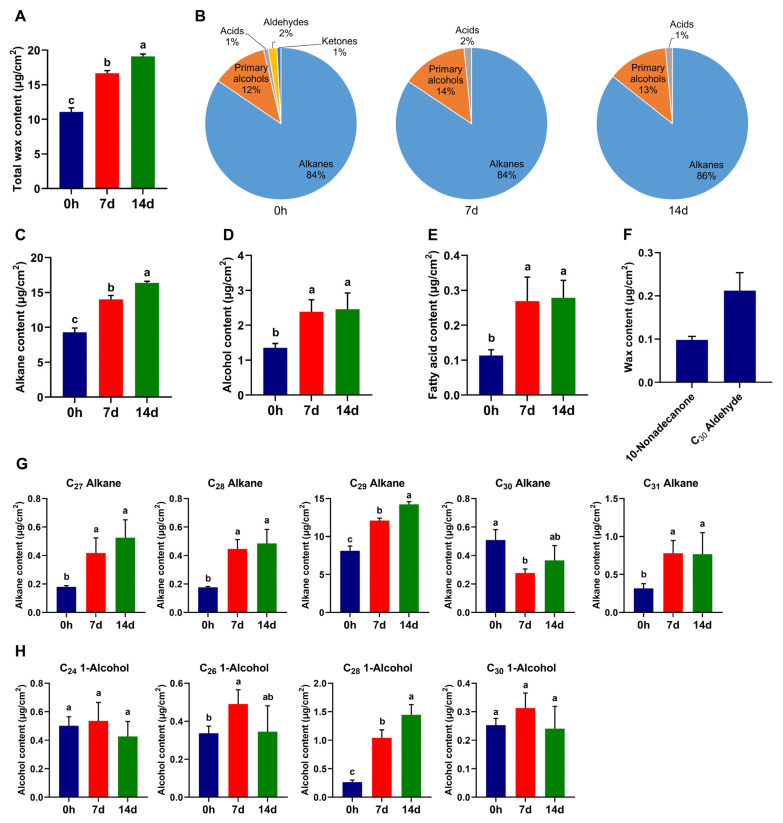
The effect of osmotic stress on the cuticular wax of *A. mongolicus* leaves. (**A**) The comparison of the total wax content between osmotic-stressed and non-stressed leaves. (**B**) The comparison of the wax composition between osmotic-stressed and non-stressed leaves. (**C**–**E**) The comparison of the different classes of wax components content between osmotic-stressed and non-stressed leaves. (**C**) Total alkanes. (**D**) Total 1-alcohols. (**E**) Total fatty acids. (**F**) The contents of aldehydes and ketones identified in non-stressed leaves. (**G**) The comparison of all alkane component content between osmotic-stressed and non-stressed leaves. (**H**) The comparison of all 1-alcolhol component content between osmotic-stressed and non-stressed leaves. LSD and DMRT were used to conduct multiple comparisons. Lowercase letters (a–c) above columns represent the different homogeneous subsets according to multiple comparisons.

GC-MS analyses revealed the content and composition of cuticular wax in non-stressed *A. mongolicus* leaves. Total wax content of leaves was 11.09 μg/cm^2^ (Figure 2A). Five classes of aliphatic compounds were identified from non-stressed leaf cuticular wax, namely alkanes, primary alcohols (1-alcohols), fatty acids, aldehydes, and ketones. Notably, alkanes constituted over 80% of the total wax content (Figure 2B–F). A total of five alkane components with different carbon chain lengths were identified, all of which are long straight-chain alkanes, and the content of nonacosane (C_29_ Alkane) accounted for 87% of the total alkanes (Figure 2G). The 1-alcohols of the wax consisted of four components with carbon chains ranging from C_24_ to C_30_ (Figure 2H). Fatty acids, aldehydes, and ketones each included only one component, namely n-triacontanoic acid (C_30_ Acid), n-triacontanal (C_30_ Aldehyde), and 10-nonadecanone (10-C_19_ Ketone) (Figure 2E,F).

### 3.3. The Alteration of Cuticular Wax of A. mongolicus Leaves under Osmotic Stress

The GC-MS analyses further revealed the alteration of wax content and composition of *A. mongolicus* leaves under osmotic stress. The total wax content gradually increased during persistent osmotic stress. Following 14-day osmotic stress, the total wax content increased to 19.11 μg/cm^2^, exhibiting a 72% increase compared to the control group (Figure 2A). Only three classes of components, alkanes, 1-alcohols and fatty acids, were identified in stressed leaf cuticular wax (Figure 2B).

Under osmotic stress, the total alkanes, 1-alcohols, and fatty acids in the cuticular wax had significantly increased contents. Compared to non-stressed leaves, the total alkane content of 7- and 14-day-osmotic-stressed leaves increased by 50% and 76%, respectively (Figure 2C). The contents of 1-alcohol increased by 75% and 81% (Figure 2D), and the content of C_30_ acid increased by 137% and 146% under 7- and 14-day stress, respectively (Figure 2E). The content of the five alkane compounds had varied degrees of development under osmotic stress, except for n-triacontane (C_30_ alkane) (Figure 2G). Among the four 1-alcohol compounds, the content of n-octacosanol (C_28_ 1-alcohol) significantly increased after 7- and 14-day osmotic stress, and the content of n-hexacosanol (C_26_ 1-alcohol) increased under 7-day stress (Figure 2H).

### 3.4. Transcriptome Analysis of the Response of A. mongolicus Leaves to Osmotic Stress

**Figure 3:**
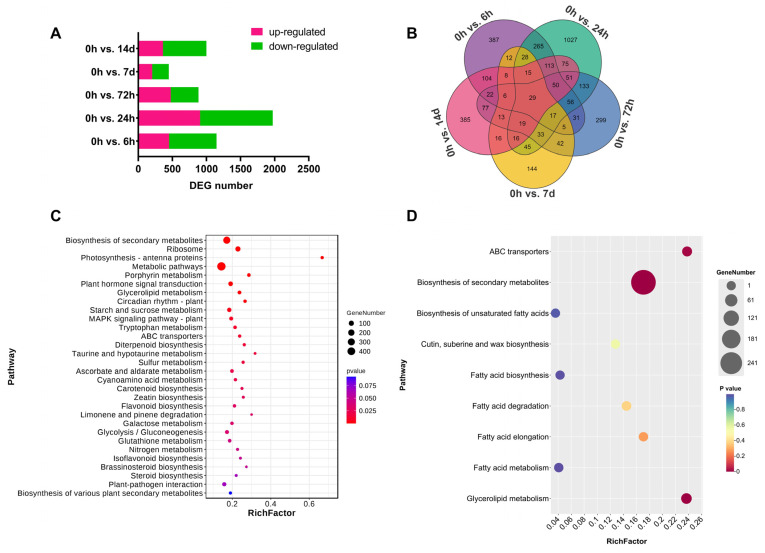
The transcriptome analysis of *A. mongolicus* leaves under osmotic stress. (**A**) The number of up-regulated and down-regulated DEGs at time points of 6 h, 24 h, 72 h, 7 d, and 14 d. (**B**) The Venn plot of the DEG number shared among different time point groups. (**C**) The KEGG pathway enrichment analysis of all unique DEGs. (**D**) The KEGG pathway enrichment analysis from all unique DEGs related to cuticular wax biosynthesis and transportation.

To investigate the dynamics of cuticular wax-related genes in the leaves of *A. mongolicus* under osmotic stress, time-course RNA-seq analyses were conducted and over 24,000 genes were characterized. Compared to the control group (0 h), a total of 1148, 1972, 883, 448, and 999 differentially expressed genes (DEGs) were identified at 6 h, 24 h, 72 h, 7 d, and 14 d time points, respectively (Figure 3A). A total of 3523 unique DEGs were detected, with 387, 1,027, 299, 144, and 385 DEGs exclusively identified at 6 h, 24 h, 72 h, 7 d, and 14 d time points, respectively. Furthermore, 29 DEGs were shared across all time points during osmotic stress (Figure 3B).

KEGG enrichment analysis for all unique DEGs demonstrated that multiple pathways, such as “Biosynthesis of secondary metabolites (ko01110)”, “Plant hormone signal transduction (ko04075)”, and “Glycerolipid metabolism (ko00561)”, were associated with osmotic stress response in *A. mongolicus* leaves (Figure 3C; Table S2). We further analyzed KEGG pathways which are associated with the accumulation of cuticular wax, including the pathways which are related to the biosynthesis of VLCFA precursors and the modification of VLCFAs to different wax components, as well as associated wax transportation mechanisms (Figure 3D; Table S2). For example, a total of six DEGs were annotated in “Fatty acid elongation (ko00062)”, and five DEGs were annotated in “Cutin, suberine and wax biosynthesis (ko00073)”.

### 3.5. Transcriptome Analysis Revealed That Cuticular Wax Biosynthesis and Transport Pathways in A. mongolicus Leaves Respond to Osmotic Stress

**Figure 4:**
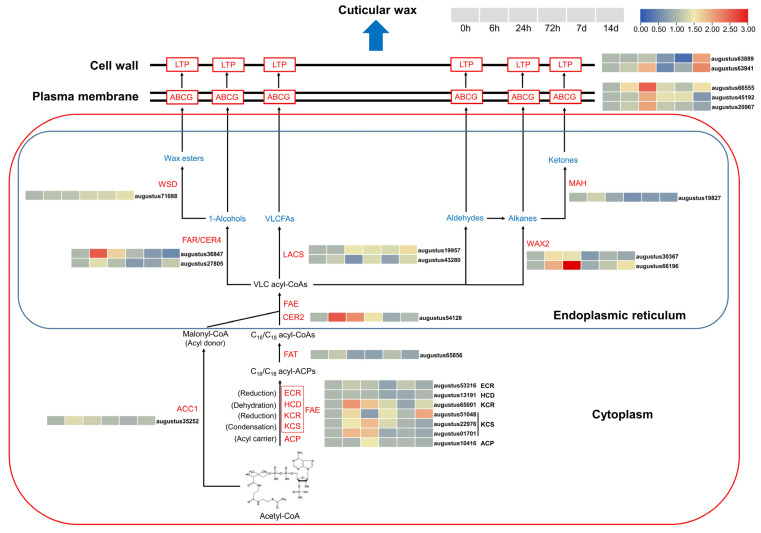
The expression patterns of functional genes involved in cuticular wax biosynthesis and transport under osmotic stress in *A. mongolicus*. Rectangles represent the abundance fold-change compared to the non-stressed (0 h) group based on FPKM values of functional genes in different time points under osmotic stress. The color scales represent higher (red) to lower (blue) abundance. The data were visualized after normalization using logarithm base 2.

We further analyzed the expression patterns of the functional genes involved in the biosynthesis and transportation pathways of cuticular wax in *A. mongolicus* leaves under osmotic stress (Figure 4). In the VLCFA biosynthesis pathway, several enzyme genes, including three β-ketoacyl-CoA synthase (KCS) coding genes (augustus01701, augustus22976, augustus51048) and one β-ketoacyl-CoA reductase (KCR) coding gene (augustus65001), whose coding proteins form the Fatty Acid Elongation complex (FAE), had increased expression under different time points of osmotic stress. Another enzyme gene, augustus54128, which codes a CER2-like protein derived from the BAHD superfamily of acyltransferases and directly catalyzes the production of VLC acyl-CoAs, had an over two-fold transcription abundance at 6 h, 24 h, and 72 h of osmotic stress compared to the non-stressed group. A long-chain acyl-CoA synthetase (LACS)-coding gene, augustus19957, participating in the final step of VLCFA synthesis, was up-regulated after 24 h of osmotic stress. In the alkane biosynthesis branch (also known as the decarboxylation pathway), a VLC-Acyl-CoA reductase gene, augustus65001 (*WAX2*), was strongly induced by osmotic stress at the 24 h time point. In the alcohol-forming pathway (also known as the acyl reduction pathway), augustus36847, which codes the enzyme directly catalyzing the production of primary alcohols, had 4.7-fold and 2.2-fold expression levels at 6 h and 24 h of osmotic stress, respectively. Additionally, altered transcriptions of some wax transporter genes were also detected. Several ATP-binding cassette transporter G (ABCG)-coding genes, such as augustus66555, augustus45192, and augustus20967, which conduct the plasma membrane transportation of wax components, had increased expression at different time points of osmotic stress. Two lipid transfer protein (LTP)-coding genes, augustus63889 and augustus63941, which code the wax transporter proteins across the cell wall, both had three-fold expression levels at the 14 d time point.

**Figure 5:**
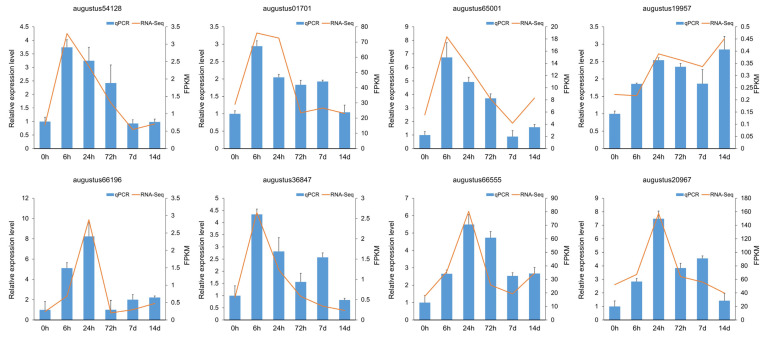
The qRT-PCR verification for RNA-seq results. The RNA-seq result of each gene was expressed as the FPKM value, while the qRT-PCR result (relative expression level) of each gene was calculated using the 2^−ΔΔCt^ method. Each assay was performed in three independent biological replicates.

To validate the results of RNA-seq, qRT-PCR was conducted on eight of the above genes (Figure 5), including six enzyme genes, namely augustus54128 (coding for CER2), augustus01701 (coding for KCS), augustus65001 (coding for KCR), augustus19957 (coding for LACS), augustus66196 (coding for WAX2), and augustus36847 (coding for fatty acyl-CoA reductase, FAR), and two ABCG transporter genes, augustus66555 and augustus20967.

### 3.6. WGCNA Analysis Uncovered Key Modules and Genes Related to Leaf Cuticular Wax Accumulation in A. mongolicus under Osmotic Stress

**Figure 6:**
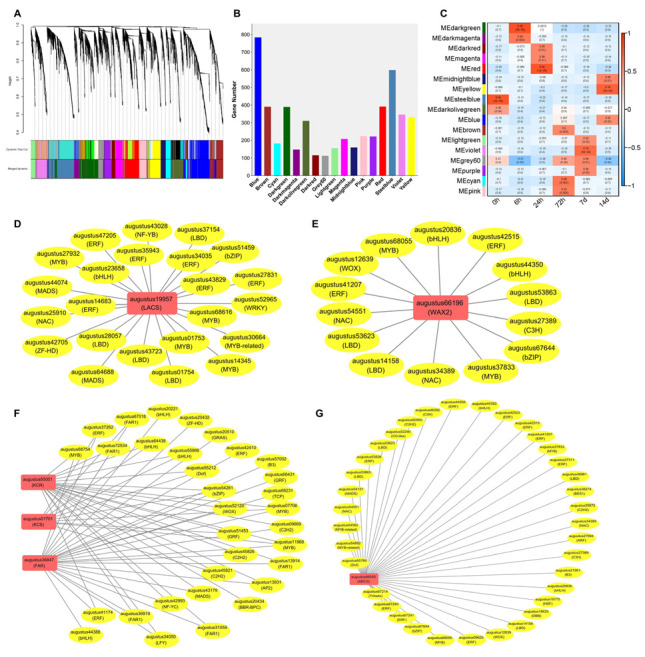
The WGCNA analysis for transcriptome data of *A. mongolicus* leaves under osmotic stress. (**A**) Clustering of genes based on topological overlap. Different colors in Dynamic Tree represent corresponding gene clusters in the above cluster tree, and different colors in Merged Dynamic represent merged gene sets with similar expression patterns. (**B**) The number of hub genes in each co-expression module. (**C**) The module–trait association analysis. Co-expression modules identified in six time-point transcriptomic samples are demonstrated. Color scale represents positive (red) to negative (blue) correlation between modules and samples. (**D**) Co-expression network identified from the MEblue module associated with the wax biosynthesis pathway with *LACS* as the hub gene. (**E**) Co-expression network identified from the MEred module associated with the wax biosynthesis pathway with *WAX2* as the hub gene. (**F**) Co-expression network identified from the MEdarkgreen module associated with the wax biosynthesis pathway with *KCR*, *KCS*, and *FAR* as the hub genes. (**G**) Co-expression network identified from the MEred module associated with the wax transportation pathway with an ABCG transporter coding gene as the hub gene. The red rounded rectangles represent the enzyme- or transporter-coding genes, and yellow ovals indicate the co-expressed TF genes.

To identify the gene co-expression modules responding to osmotic stress in *A. mogolicus* leaves, the weighted gene co-expression network analysis (WGCNA) was conducted using the transcriptome data containing six time points. Expression data from 10,698 genes (FPKM ≥ 0.005) were utilized for the analysis. Through hierarchical clustering, a total of 17 co-expression modules were identified, which contain 5057 genes. The MEblue module was the largest, encompassing 783 genes, while the MEgrey60 module was the smallest, containing 112 genes (Figure 6A,B). Module–trait association analysis revealed the correlation between co-expression modules and transcriptome data of different time points. For example, the MEdarkgreen and MEdarkmagenta modules exhibited positive correlations with the 6 h group, the MEred and MEdarkred modules correlated positively with the 24 h group, and the MEyellow and MEblue modules showed a positive correlation with the 14 d group (Figure 6C).

We further explored key genes related to cuticular wax biosynthesis and transportation pathways and regulators associated to these genes in co-expression modules, and six hub genes were identified. In the MEblue module, augustus19957, annotated as *LACS*, emerged as a hub gene. This hub gene exhibited close associations with 23 transcription factor (TF) genes across 11 families, such as ERF, LBD, and MYB (Figure 6D). Another hub gene, augustus66196, was identified from the MEred module, and annotated as the alkane synthesis-related gene *WAX2*. This hub gene was closely associated to 15 TF genes which belong to eight families such as ERF, bHLH, and NAC (Figure 6E). A total of three hub genes from the MEdarkgreen module were identified as VLCFAs and primary alcohol synthesis-related enzyme genes, namely *KCR* (augustus65001), *KCS* (augustus01701), and *FAR* (augustus36847). These hub genes were associated with 32 TF genes belonging to 18 families, such as FAR1, MYB, and bHLH (Figure 6F). Furthermore, an ABCG transporter gene, augustus66555, was identified as a hub gene from the MEred module, and 36 TF genes across 19 families were detected as co-expressing with this ABCG gene (Figure 6G).

## 4. Discussion

As a relict genus which has only two species surviving to the present, the *Ammopiptanthus* genus has thrived in the harsh deserts of Central Asia, maintaining a perennial life cycle in adverse environments. *Ammopiptanthus* species have demonstrated strong tolerance to multiple abiotic stresses, such as drought, extreme temperatures, and intense radiation [35]. Similar to other associated shrubs in the same habitat, like *Tetraena mongolica*, *Zygophyllum xanthoxylum*, and *Nitraria tangutorum*, *A. mongolicus* exhibits morphological features supporting stress tolerance, such as well-developed underground root traits and vascular tissues with a high degree of lignification [29]. Furthermore, *A. mongolicus* has typical broad leaves with a larger leaf area index (LAI), which is a significant distinction from the above associated shrubs, suggesting that the aboveground parts of this plant confront more radiation, heat, and intense water loss during their life cycle. Thus, it is speculated that the organs and tissues of *A. mongolicus* have evolved specific physiological and metabolic mechanisms to resist these abiotic stresses and maintain optimum bioprocesses. In the present study, we conducted osmotic treatment on *A. mongolicus* seedlings. The growth assays demonstrated that the growth condition of seedlings under 7 days of continuous stress had non-significant difference from that of the non-stressed group and the P_n_ rate was also comparable to that of non-stressed seedlings (Figure 1A,B,G). These results suggested that osmotic stress has not yet imposed inhibitory effects on *A. mongolicus* seedlings during 7 days of stress, indicating that the seedlings possessed the tolerance to osmotic stress which exhibited less water loss.

The aboveground parts of terrestrial plants, primarily the leaves, are constantly involved in the process of releasing internal moisture (in the form of gas) into the external environment, i.e., transpiration, which provides the driving force for plants to absorb water and minerals, also serving to cool the leaves [36]. However, under stress conditions such as drought, high salinity, and intense radiation, increased transpiration leads to excessive water loss in plants, further suppressing optimum metabolic processes. The plant transpirational water loss process occurs through two pathways: stomatal and non-stomatal transpiration [37]. Stomatal transpiration involves the alteration of stomatal cell morphology on leaf surfaces, which creates gaps for moisture to leave the plants [38]. In the present study, the measurement of the gas exchange index revealed that the T_r_ and G_s_ of *A. mongolicus* leaves significantly decreased under osmotic stress (Figure 1H,I), indicating that seedlings reduced stomatal transpiration in response to stress. Non-stomatal transpiration refers to direct water loss through the epidermis during stomatal closure, and the epidermal permeability determines the degree of water loss rate [39]. The physiological measurements related to epidermal permeability in this study demonstrated that the water loss rate and the chlorophyll efflux rate of *A. mongolicus* leaves under osmotic stress, which were subjected to dark treatment to close stomata before measurement, were significantly lower than those of non-stressed leaves (Figure 1E,F). These results indicated that non-stomatal transpiration in leaves decreased due to lower epidermal permeability in response to osmotic stress. Previous studies reported that the alteration in plant epidermal permeability was primarily influenced by the hydrophobic cuticle layer covering epidermal cells, and the hydrophobicity of the cuticle layer was mainly supported by cuticular wax [8]. Therefore, we further analyzed the changes in the cuticular wax of *A. mongolicus* leaves to investigate factors influencing epidermal permeability.

To cope with the complex environmental changes, especially drastic fluctuations in water availability, terrestrial plants have evolved composite lipid structures including the cuticular wax layer which cover the surfaces of plant tissues [40]. The cuticle lipids, in conjunction with polysaccharide-like cellulose and pectin in the cell wall as a continuum structure, form the first physical barrier to prevent excessive water loss [8]. In the present study, cuticular wax of *A. mongolicus* leaves was extracted using organic solvents. The measurement of total wax revealed that the content in non-stressed leaves was 11.09 μg/cm^2^ (Figure 2A). Compared with reported model plants and crops, the wax content in *A. mongolicus* leaves was approximately eight-fold of that of *Arabidopsis* leaves [41] and about twice that of rice and wheat leaves [42,43]. These results suggested that *A. mongolicus*, as a shrub adapted to desert, had a higher level of wax accumulation compared to plants thriving in mild environments, providing important support for environment adaptation through cuticle lipid structures. In comparison with associated shrubs inhabiting the deserts, the wax content in *A. mongolicus* leaves was about 60% higher than that of *T. mongolica* leaves but significantly lower than that of *Z. xanthoxylum* [29]. Previous morphological observations demonstrated that *T. mongolica* had succulent rod-shaped leaves with lower LAI [29], and we speculated that *A. mongolicus*, with its broadleaf characteristics, maintained water retention through a higher wax accumulation level. Meanwhile, previous electron microscopy observations revealed the absence of trichomes on the epidermis of *Z. xanthoxylum* leaves [29]. We speculated that *A. mongolicus*, like *T. mongolica* possessing both trichomes and a wax layer, jointly provide structural support for water retention in leaves. Under osmotic stress, the total cuticular wax content of *A. mongolicus* leaves was significantly increased, indicating the activation of wax accumulation-related pathways and thicker wax layer to reduce epidermal permeability, thereby inhibiting water loss induced by osmotic stress.

GC-MS analysis revealed that the cuticular wax of *A. mongolicus* leaves consists of VLCFAs and derivatives including alkanes, 1-alcohols, aldehydes, and ketones (Figure 2B). The alkane content constitutes over 80% of total wax, and the number of alkane components accounts for half of all wax components (Figure 2B,G), indicating that long straight-chain alkanes are the primary components forming the wax layer of leaves. Similar results were reported for associated shrubs, *T. mongolica*, and *Z. xanthoxylum* [29]. In contrast, the cuticular wax of rice and wheat leaves is primarily composed of 2-alcohols [42,43]. Another wax analysis for five *Dianthus* species revealed that C_28_ 1-alcohol and diketones were the major components forming the platelet-shaped and the tubular wax crystal, respectively [44]. The major component of cuticular wax in *T. salsuginea* rosette leaves was fatty acid [45,46]. The straight-chain alkanes are characterized by the “C-C” single bonds without side chains and modified groups, which contribute to strong hydrophobicity [27]. It is speculated that cuticular wax, primarily composed of alkanes, provides hydrophobic support to the cuticle layer of *A. mongolicus* leaves, enabling this plant to survive in a water-deficient desert. Under osmotic stress, the content of all alkane components significantly increased except for C_30_ alkane (Figure 2G), indicating that alkanes are the primary components in wax metabolism responding to osmotic stress. Previous studies demonstrated that multiple plant species such as *Arabidopsis*, *Poa pratensis*, and alfalfa exhibited significant increase in the alkane content of cuticular wax, further leading to the reduction in epidermal permeability under drought conditions [27,47,48]. Furthermore, the present study revealed that alkanes with odd carbon chains, like C_29_ alkane, were major components within increased alkanes in *A. mongolicus* under osmotic stress (Figure 2G). Similar results were reported from wax analyses of *T. mongolica*, *Z. xanthoxylum*, and *T. salsuginea*, with C_29_ alkane and C_31_ alkane being major components [29,46]. Another report for cuticular wax of different tissues in rose revealed that leaf wax with higher hydrophobicity primarily consisted of saturated straight-chain alkanes ranging from C_29_ to C_33_, while the less hydrophobic petal wax mainly included saturated alkanes from C_25_ to C_27_ and unsaturated alkanes [49]. Thus, it is suggested that the increased wax components, especially the alkanes like C_29_ alkane, enhanced the hydrophobicity of the wax layer on the leaf epidermis in *A. mongolicus*, thereby suppressing water loss induced by osmotic stress.

The KEGG enrichment analysis of differentially expressed genes (DEGs) identified in the present transcriptome data demonstrated that multiple metabolic pathways and biological processes responded to osmotic stress, including several pathways directly related to cuticular wax metabolism (Figure 3C,D; Table S2). In addition, some pathways were identified as the orchestras of these metabolites. For example, the present study revealed that some plant hormone signal transduction pathways were enriched under osmotic stress (Figure 3C; Table S2), such as abscisic acid (ABA) and jasmonic acid (JA). ABA and JA were reported as key regulators of abiotic stress response in plants, which participated in the metabolism of multiple secondary metabolites such as lignin, flavonoids, anthocyanins, and terpenes [50,51,52]. In recent years, the plant cuticular wax metabolisms involved in ABA and JA signal transduction were revealed. For instance, exogenous ABA treatment induced the accumulation of long straight-chain alkanes like hentriacontane (C_31_ alkane) [53]. Exogenous JA methyl ester (MeJA) treatment led to an increase in the amounts of aldehydes and ketones in *Brassica napus* leaves [54]. Two MYB TFs in *Arabidopsis*, AtMYB94 and AtMYB96, had an ABA-dependent additive influence on wax biosynthesis by directly regulating key enzyme genes such as *KCS6* and *CER2* [55]. *ZmGL8*, which encodes a 3-ketoacyl reductase in maize, was involved in JA-mediated wax production [56]. In the present study, some genes associated with biosynthesis and signal transduction of ABA and JA were up-regulated under osmotic stress, such as augustus68437 (encoding an ABA synthesis-related enzyme, NCED), augustus53474 (encoding an ABA receptor protein, PYR), augustus01705 (encoding MYC TF), and augustus11611 (encoding a JA synthesis-related enzyme, OPR) (Table S2). It is speculated that the ABA and JA signal pathway orchestrated the process of wax accumulation in *A. mongolicus* under osmotic stress.

The biosynthesis of plant cuticular wax involves a complex metabolic network with multiple branch pathways and various functional genes [12]. In this study, we analyzed the expression profiles of genes associated with wax biosynthesis pathways using time-course transcriptomic data of *A. mongolicus* leaves under osmotic stress. The precursors for cuticular wax production are C_16_ and C_18_ saturated fatty acids, which are synthesized in the cytoplasmic matrix. Their synthetic reactions, utilizing acetyl-CoA as the substrate, are catalyzed by a FAE complex comprised of four enzymes: β-ketoacyl-CoA synthase (KCS), β-ketoacyl-CoA reductase (KCR), β-hydroxyacyl-CoA dehydratase (HCD), and enoyl-CoA reductase (ECR). Through a series of condensation–reduction–dehydration–reduction reaction cycles catalyzed by these enzymes, the acyls carried by the acyl carrier protein (ACP) are added to the carbon chain until the C_16_ and C_18_ saturated acyl-ACPs formed. Subsequently, free C_16_ and C_18_ fatty acyl-CoAs are released through hydrolysis by fatty acyl-ACP thioesterase (FAT) and transported to the ER for further VLCFA synthesis, which is also catalyzed by FAE [12]. The transcriptomic analyses in the present study unveiled some up-regulated genes which encoded enzymes of FAE, such as augustus51048 (coding for KCS) and augustus65001 (coding for KCR), at several time points under osmotic stress (Figure 4). Previous studies have identified these enzymes in multiple plant species. For example, overexpression of *MsKCS10* promoted the accumulation of cuticular wax in alfalfa leaves and enhanced drought resistance [57]. Additionally, another report revealed that three CER2 proteins participating in FAE played a crucial role in VLCFA synthesis, especially for the carbon chain above C_28_ [58]. Present transcriptomic analyses indicated the significant increase in the expression of a gene encoding a CER2-like protein, augustus51048, at time points of 6, 24, and 72 h (Figure 4). These results suggested that osmotic stress induced the activation of the VLCFA synthesis pathway in *A. mongolicus*. In ER, the VLC acyl-CoAs are further transformed into various wax components through two main pathways: the decarboxylation and the acyl reduction pathway. In the decarboxylation pathway, VLC acyl-CoAs are converted into alkanes catalyzed by WAX2 [12]. A study on cucumber cuticular wax biosynthesis revealed that the cucumber line overexpressing *CsWAX2* had increased contents of total alkanes and total cuticular wax, and higher resistance to water deficit and pathogens [59]. Present transcriptomic analyses unveiled that the transcription of a gene encoding WAX2, augustus66196, was induced by osmotic stress (Figure 4), suggesting more alkane transformation in *A. mongolicus* leaves under osmotic stress. This result was consistent with accumulated alkane components according to the wax analyses. In a word, present transcriptomic analyses indicated that wax biosynthesis including VLCFAs and alkanes was activated in *A. mongolicus* leaves under osmotic stress.

The wax components synthesized in epidermal cells need to be transported across the plasma membrane and the cell wall so as to be secreted onto the plant tissue surface. Previous studies revealed that the ABC transporters, especially the ABCG subfamily, played important roles in transporting wax components across the plasma membrane [60,61]. Some ABCG members involved in abiotic stress response were also isolated. For instance, ectopic expression of *TsABCG11* (isolated from *T. salsuginea*) in *Arabidopsis* resulted in increased cuticular wax and cutin content, along with enhanced tolerance to salt and cold stress [62]. The rice *abcg9* mutant exhibited a half reduction in wax content and decreased resistance to drought stress [63]. Transcriptomic analyses in this study revealed that several ABCG genes, such as augustus66555 and augustus45192, were up-regulated under osmotic stress (Figure 4). It is speculated that the transmembrane transportation of wax in *A. mongolicus* leaves was more active due to the up-regulation of ABCG transporters. Previous studies suggested that a class of small, basic proteins containing a cell wall-targeting signal peptide known as LTPs were involved in the transport of wax components across the cell wall [64,65]. In this study, LTP-coding genes such as augustus63941 showed up-regulated abundances under osmotic stress (Figure 4), indicating more activated involvement of LTPs in transporting wax components. These results suggested, in addition to biosynthesis, that the transport pathways were also activated to promote wax accumulation in *A. mongolicus* under osmotic stress.

## 5. Conclusions

**Figure 7:**
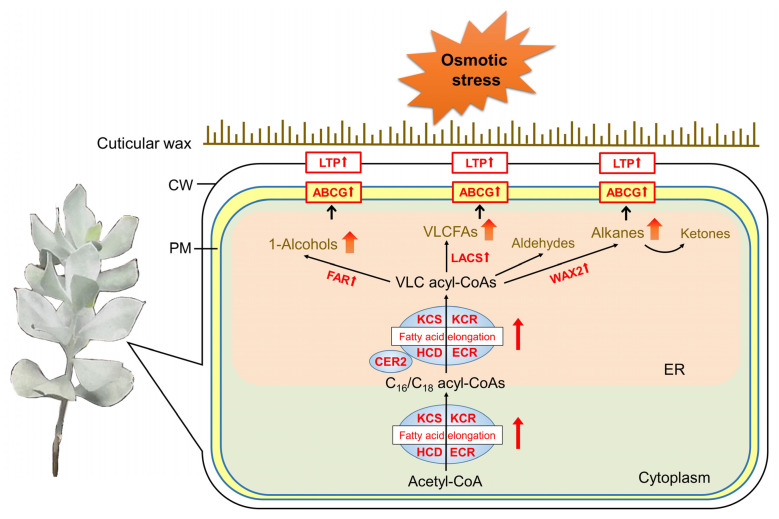
A graphic overview of leaf cuticular wax metabolism under osmotic stress in *A. mongolicus*. The black arrows represent biosynthesis reactions and transportation of cuticular wax. The red thicken arrows beside the enzyme and transporter proteins represent the un-regulation of these proteins. The orange thicken arrows beside the wax components represent the increase of these metabolites. Under osmotic stress, the up-regulated enzyme genes constituting the FAE complex catalyzed the synthesis of increased VLC acyl-CoAs, which provided more precursors for wax components. In decarboxylation and acyl reduction pathways, the production of alkanes and 1-alcohols was more activated due to the up-regulation of FAR and WAX2, respectively. Furthermore, more activated ABCG transporters and LTPs successively promoted wax transport across the plasma membrane and the cell wall, and ultimately led to the accumulation of the wax layer in the leaf cuticle. ER: endoplasmic reticulum; PM: plasma membrane; CW: cell wall.

In the present study, alterations of cuticular wax and related gene expression in the leaves of *A. mongolicus* under osmotic stress were investigated. The epidermal permeability of leaves decreased, and transpirational water loss was inhibited under osmotic stress. A total of five classes of components were identified from the leaf cuticular wax, with long straight-chain alkanes being the major components. The content of total cuticular wax, multiple alkane and primary alcohol components significantly increased under osmotic stress. The expression levels of multiple genes involved in the synthesis of VLCFAs and alkanes, as well as the those responsible for cuticular wax transport, were significantly up-regulated (Figure 7). Some co-expressed modules associated with cuticular wax synthesis and transport pathways were identified, and six hub genes were identified as the key regulators for wax metabolism, including enzyme genes such as *KCS* and *WAX2*, and an ABCG gene.

## Data Availability

All data supporting this study are available within the paper and within the supplementary materials published online. The transcriptomic data used in the present study were submitted to NCBI SRA with accession number range from SRR26384053 to SRR26384070 (included in Bioproject: PRJNA1027962).

## Supplementary Materials

The following supporting information can be downloaded at: https://www.mdpi.com/article/10.3390/biom14020227/s1, Table S1: The sequences of primers for qRT-PCR analyses. Table S2: The DEGs enriched in different KEGG pathways.
